# Whole-Body Functional MRI and PET/MRI in Multiple Myeloma

**DOI:** 10.3390/cancers12113155

**Published:** 2020-10-27

**Authors:** Sébastien Mulé, Edouard Reizine, Paul Blanc-Durand, Laurence Baranes, Pierre Zerbib, Robert Burns, Refaat Nouri, Emmanuel Itti, Alain Luciani

**Affiliations:** 1 SyMPTOm PET/MRI Platform, Henri Mondor Hospital, AP-HP, 94010 Créteil, France; ereizine@gmail.com (E.R.); paul.blancdurand@aphp.fr (P.B.-D.); laurence.baranes@aphp.fr (L.B.); pierre.zerbib@aphp.fr (P.Z.); robert.burns@outlook.fr (R.B.); refaat.nouri@gmail.com (R.N.); emmanuel.itti@aphp.fr (E.I.); alain.luciani@aphp.fr (A.L.); 2 Department of Medical Imaging, Henri Mondor Hospital, AP-HP, 94010 Créteil, France; 3 Université Paris-Est Créteil, 94010 Créteil, France; 4 Department of Nuclear Medicine, Henri Mondor Hospital, AP-HP, 94010 Créteil, France

**Keywords:** multiple myeloma, MRI, functional imaging, PET/MRI, whole-body imaging

## Abstract

**Simple Summary:**

Whole-body magnetic resonance imaging (MRI) is recognized as the most sensitive imaging technique for the detection of bone marrow infiltration, and was therefore, recently included in the new diagnostic myeloma criteria, as proposed by the International Myeloma Working Group. The use of diffusion-weighted MRI further improved the performances of whole-body MRI in the setting of multiple myeloma, and its systematic implementation in general clinical practice is now recommended. Whole-body, dynamic, contrast-enhanced MRI might provide further information on lesions vascularity and might help evaluate response to treatment. Hybrid PET/MRI might act as the optimal imaging modality, owing to the association of the best techniques for both detecting bone marrow involvement and evaluating treatment response, providing one-stop-shop imaging in a whole-body scale. This review provides an overview on the value of whole-body MRI, including diffusion-weighted and dynamic contrast-enhanced MRI and whole-body ^18^F-FDG PET/MRI in diagnosis, staging, and response evaluation in multiple myeloma.

**Abstract:**

Bone disease is one of the major features of multiple myeloma (MM), and imaging has a pivotal role in both diagnosis and follow-up. Whole-body magnetic resonance imaging (MRI) is recognized as the gold standard for the detection of bone marrow involvement, owing to its high sensitivity. The use of functional MRI sequences further improved the performances of whole-body MRI in the setting of MM. Whole-body diffusion-weighted (DW) MRI is the most attractive functional technique and its systematic implementation in general clinical practice is now recommended by the International Myeloma Working Group. Whole-body dynamic contrast-enhanced (DCE) MRI might provide further information on lesions vascularity and help evaluate response to treatment. Whole Body PET/MRI is an emerging hybrid imaging technique that offers the opportunity to combine information on morphology, fat content of bone marrow, bone marrow cellularity and vascularization, and metabolic activity. Whole-body PET/MRI allows a one-stop-shop examination, including the most sensitive technique for detecting bone marrow involvement, and the most recognized technique for treatment response evaluation. This review aims at providing an overview on the value of whole-body MRI, including DW and DCE MRI, and combined whole-body ^18^F-FDG PET/MRI in diagnosis, staging, and response evaluation in patients with MM.

## 1. Introduction

Multiple myeloma (MM) is a hematological disorder characterized by the proliferation of plasma cells producing abnormal monoclonal immunoglobulin and infiltrating bone marrow [1]. Bone involvement is one of the most prominent features of MM, and imaging has an increasingly important role in the diagnosis, initial staging, and follow-up of MM patients. Other major clinical manifestations include anemia, hypercalcemia, and renal failure (CRAB criteria).

Conventional skeletal survey was historically used for the assessment of bone lesions in patients with MM, due to both low costs and widespread availability. However, cross-sectional imaging including whole-body CT, PET/CT, and whole-body magnetic resonance imaging (MRI) showed higher sensitivity for the detection of focal lesions.

Among them, whole-body MRI is the most sensitive technique for the detection of bone marrow involvement and allows the detection of both diffuse bone marrow infiltration and focal lesions, before the mineral bone is destroyed [2]. This has led the International Myeloma Working Group to recommend whole-body MRI as first-line imaging for all patients with a suspected diagnosis of asymptomatic myeloma or solitary bone plasmacytoma [3]. Moreover, MRI is the modality of choice for differentiating benign from malignant vertebral fractures, and for assessing painful complications and spinal cord compression [3]. Moreover, both focal and diffuse MRI findings were shown to have prognostic significance.

The use of functional MRI sequences further improved the performances of whole-body MRI in the setting of MM. Whole-body diffusion-weighted (DW) MRI is the most attractive functional technique and was shown to be superior to PET/CT for bone marrow involvement detection [4]. Its systematic implementation in general clinical practice is now recommended by the International Myeloma Working Group [5]. Whole-body dynamic contrast-enhanced (DCE) MRI might provide further information on lesions vascularity and help evaluate response to treatment [6]. This increasingly important role of whole-body MRI has led to the design of Myeloma Response Assessment and Diagnosis System (MY-RADS) imaging recommendations, on the standards for acquisition protocol and analysis methods [7]. Again, whole-body DW MRI is recommended to be systematically performed.

Whole-body MRI including functional imaging techniques, thus, provides different information from that offed by PET. The recent emergence of hybrid PET/MRI scanners offers the opportunity to combine information on bone marrow cellularity and vascularization, and metabolic activity. PET/MRI allows a one-stop-shop examination, including the most sensitive technique for detecting bone marrow involvement and the most recognized technique for treatment response evaluation [8].

This review aims at providing an overview on the value of whole-body MRI, including DCE MRI and DW MRI, and combined whole-body ^18^F-FDG PET/MRI in diagnosis, staging, and response evaluation in patients with MM.

## 2. Clinical Protocol for Whole-Body MRI

### 2.1. Conventional MR Imaging Findings

Bone marrow might be focally or diffusely involved throughout the body in patients with MM. Therefore, wide MRI anatomic coverage is necessary for an accurate assessment of disease burden. Indeed, up to half of lesions might be missed when imaging the spine only [9]. Therefore, MRI spatial coverage should include the axial skeleton and the proximal appendicular skeleton. Such a large anatomic scale, high resolution MRI examination within a reasonable imaging time was achieved through the combination of a rolling-table platform, multichannel phased-array surface coils, and parallel imaging [10].

Conventional MR sequences include T1-weighted spin-echo and fat-suppressed T2-weighted sequences. The whole spine is covered in the sagittal plane, while the skull bone, the ribs, the pelvis and the proximal appendicular skeleton are covered in either the transverse or the coronal plane. The recommended section thickness is 4–5 mm (as a positive lesion is considered a lesion of diameter ≥5 mm). In our institution, whole-body MRI examinations are performed either with a 1.5-T MR system (Magnetom Avanto fit; Siemens Healthineers, Erlangen, Germany) or a 3T integrated PET/MRI system (Biograph mMR; Siemens Healthineers, Erlangen, Germany). T1-weighted spin-echo sequences are acquired in the sagittal plane covering the whole spine, and in the coronal plane, covering the pelvis and proximal femurs. Dixon T2-weighted water-only sequences are also acquired in the sagittal plane covering the whole spine, and in the coronal plane covering from the neck to proximal femurs. The mean acquisition time of conventional MR images in our institution is around 15 min.

Multiple myeloma focal lesions typically have low signal intensity in T1-weighted MR images, due to low fat content, and rather high signal intensity in fat-suppressed T2-weighted and STIR MR images. Focal lesions should have a diameter ≥5 mm. Focal lesions are more frequently seen in the axial skeleton, with a predominance in the thoracic and lumbar spine, which correspond to the sites containing more red bone marrow. Extra-axial focal lesions are predominantly found in proximal femurs and humerus.

**Figure 1:**
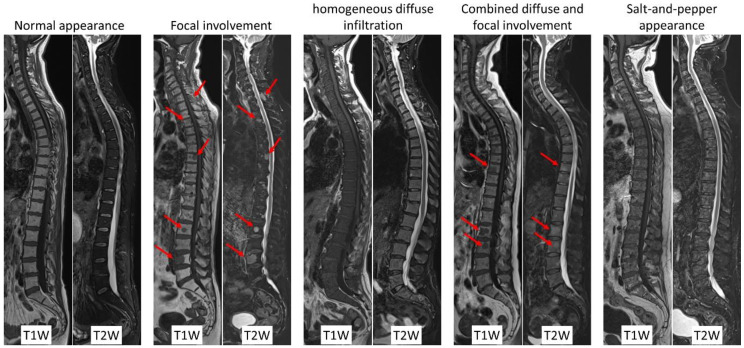
T1-weighted spin-echo and Dixon T2-weighted water-only images of the whole spine in the sagittal plane. Typical MRI findings associated with the five recognized bone marrow infiltration patterns. From left to right—normal appearance of bone marrow, focal lesion or focal involvement (arrows), homogeneous diffuse infiltration, combined diffuse and focal (arrows) involvement, and variegated or salt-and-pepper appearance.

Bone marrow infiltration might have different patterns, five of them were described and are recognized—normal appearance of bone marrow, focal lesion or focal involvement, homogeneous diffuse infiltration, combined diffuse and focal involvement, and variegated or salt-and-pepper appearance (Figure 1). High tumor burden is typically suspected in the case of focal or diffuse bone marrow involvement [3]. Bone marrow diffuse infiltration is characterized by diffuse low signal intensity in T1-weighted MR images, which might be difficult to diagnose when the infiltration is moderate. In case of high-grade diffuse bone marrow involvement, the signal intensity becomes equal to or lower than that of the intervertebral discs, which should be used as a reference [5].

Conventional MRI revealed more extensive disease in half of the patients compared to whole-body CT [11]. Compared to ^18^F-FDG PET/CT, whole-body MRI has a significantly higher sensitivity, revealing bone marrow involvement—mainly diffuse infiltration—in areas of the spine and pelvis, without ^18^F-FDG PET/CT abnormal findings in up to 30% of patients, according to Zamagni et al. [12]. Moreau et al. found high sensitivity (>90%) for both methods without any significant difference between them [13].

The five above mentioned recognized infiltration patterns were shown to have an independent prognostic value. Indeed, the presence of more than 7 focal lesions was found to be an independent predictor of both event-free survival and overall survival (hazard ratio, 1.89 [1.30–2.75]) [14] in myeloma patients, while the presence of more than one focal lesion was an independent adverse prognostic factor for progression into symptomatic MM in patients with asymptomatic MM [15]. Moderate and severe diffuse bone marrow infiltration were also found to adversely affect survival [16,17]. Nonetheless, the Durie-Salmon PLUS staging system considers the number of focal lesions but not the homogeneous or salt-and-pepper diffuse patterns [18].

Conventional MRI might help to evaluate the response to therapy. Indeed, post-treatment changes of the MRI patterns occurs and are correlated to treatment response [19]. However, specificity is poor and the rate of false positive findings is high, owing to the potential persistence of nonviable focal lesions. ^18^F-FDG PET/CT was found to have a greater prognostic value at both baseline and before transplantation [20]. After chemotherapy, bone marrow hyperplasia might occur and could also lead to false-positive results; however, this effect seems to be less prominent with MRI than with PET/CT [5].

### 2.2. Whole-Body Functional MRI

To overcome the limitations of conventional MRI in both lesion detection and treatment response evaluation, imaging techniques such as diffusion-weighted imaging and dynamic contrast-enhanced magnetic resonance imaging can be used [21,22,23], providing imaging biomarkers for both prognosis and treatment response.

### 2.3. Whole-Body Diffusion-Weighted Imaging

Diffusion-weighted imaging is an MRI technique measuring the Brownian motion of water molecules. The use of diffusion gradient pulses results in MRI signal attenuation of those variations, where the gradient pulses profiles are routinely assessed by the apparent diffusion coefficient (ADC). ADC inversely correlated with cell density, as water diffusion typically slows down when tumor cellularity increases. Low ADC values were found to be associated with high degree of malignancy in various cancers [24].

However, interpretation of DW imaging findings in bone marrow differs from that in soft tissues. Indeed, normal bone marrow has very low ADC values, ranging from 0.2 to 0.5 × 10^−3^ mm^2^/s in vertebral bodies, owing to low water content, presence of large hydrophobic lipid cells, low vascularity, and reduced extracellular space [25]. Bone marrow lesions in MM appear as areas of increased signal intensity on both low (≤100 s/mm^2^) and high (500–1000 s/mm^2^) *b* value DW images, compared to normal bone marrow, due to higher cellularity and vasculature and less fat content. The lesion ADC values would be increased compared to those in normal marrow. Koutoulidis et al. found significant different ADC values between patients with normal, diffuse, and focal MR imaging patterns, with respective mean ADC values of 0.360 × 10^−3^ mm^2^/s, 0.770 × 10^−3^ mm^2^/s and 1.046 × 10^−3^ mm^2^/s [26].

**Figure 2:**
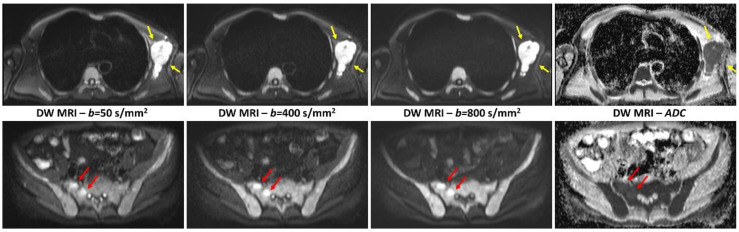
Transverse diffusion-weighted images acquired with three different *b* values (from left to right: 50 s/mm^2^, 400 s/mm^2^ and 800 s/mm^2^) and the corresponding ADC images in a 76-year-old man with bone marrow focal lesions in the pelvis (**red** arrows) and left axillary extramedullary disease (**yellow** arrows). Bone marrow diffuse involvement appears as diffuse high signal intensity areas on both low and high *b* values diffusion-weighted images, with increased ADC values (mean 0.420 × 10^−3^ mm^2^/s). Both signal intensity on high *b* value diffusion-weighted images and ADC values (0.620 × 10^−3^ mm^2^/s in one pelvic lesion and 0.710 × 10^−3^ mm^2^/s in the axillary lesion) are highest in the focal lesions.

In our institution, DW MRI sequence is based on single-shot, spin-echo,, echo-planar imaging with parallel imaging (generalized autocalibrating partially parallel acquisition [GRAPPA] factor of two) and spectrally selective fat saturation; six-station whole-body DW MRI is acquired in the transverse plane from the vertex to mid-thigh) at a section thickness of 5 mm, with three different *b* values (50 s/mm^2^, 400 s/mm^2^ and 800 s/mm^2^), with free breathing. Respiratory gating is added for stations covering chest and abdomen. ADC values are calculated from those three *b* values with monoexponential fitting and an ADC map is systematically generated (Figure 2). The mean acquisition time of DW images in our institution is around 15 min.

Diffusion-weighted imaging is the most sensitive sequence for the detection of bone marrow lesions, and highlight a higher sensitivity than ^18^F-FDG PET/CT [4], especially in patients with a low percentage of plasma cells [27], suggesting that diffusion-weighted imaging might enable bone marrow infiltration to be monitored in a non-invasive, quantitative way. As a result, its systematic implementation in general clinical practice is now recommended by both the International Myeloma Working Group and MY-RADS [5,7].

However, diffusion-weighted imaging lacks specificity and increased signal intensity findings on diffusion-weighted imaging should be interpreted jointly with those of anatomic images, including T1-weighted images, fat-suppressed T2-weighted images, and even CT images, if necessary [28].

After treatment, an initial increase in mean ADC values of the whole skeleton was found in responders but not in nonresponders [29]. Interestingly, a delayed fall in ADC values (at 20 weeks after chemotherapy) was observed by Messiou et al., which might reflect delayed return of marrow fat in lesions [30]. Beyond percentage change in mean ADC values, histogram analysis of ADC values might also help evaluate response to treatment. ADC histogram after treatment show displacement to the right or flattening, potentially indicating decreased cellularity and indicating return of marrow fat, while ADC histograms before and after treatment in nonresponders show little change in both position or shape [29]. The IMWG recently recommended that whole-body MRI should include DW imaging for treatment response assessment, in the case of PET/CT unavailability [5].

Beyond ADC, the intravoxel incoherent motion (IVIM) model attributes DWI signal changes in the low *b* value range to tissue microcapillary perfusion, while signal changes in the high *b* value range reflect true molecular diffusion. IVIM DWI might allow better understanding of diffusion-related behavior of bone marrow involvement in MM [31]. Bourillon et al. found that increased angiogenesis in focal lesions was associated with high perfusion fraction *f*, while true molecular diffusion *D* was significantly increased in diffuse bone marrow involvement [32]. Hence, IVIM DWI was suggested to be an ideal technique when the use of DCE MRI is limited due to impaired kidney function. Moreover, both true molecular diffusion *D* and perfusion fraction *f* were found to be of prognostic value, with significantly different values between early and advanced stages of newly diagnosed MM [33]. After treatment, the decreased maximal enhancement value of focal lesions in good responders is generally associated with decreased perfusion fraction.

### 2.4. Whole-Body Dynamic Contrast-Enhanced MRI

Along with DWI MRI, DCE MRI might provide functional information on bone marrow involvement in MM. Dynamic MRI consists of the acquisition of serial images before and after the intravenous injection of a gadolinium chelate. In our institution, whole-body, three-station dynamic, three-dimensional, fat-saturated volumetric interpolated breath-hold examination (VIBE) using two-point Dixon fat–water separation and controlled aliasing in parallel imaging, results in higher acceleration (Dixon-VIBE-CAIPIRINHA) images in the coronal plane with an isotropic spatial resolution of 2.3 mm.

Each acquisition requires three table movements and has a temporal resolution of 39 s. A bolus of 0.2 mL/kg of gadoterate meglumine (Dotarem; Laboratoire Guerbet, Aulnay-sous-Bois, France) is administrated at a rate of 2 mL/s, before acquisition of the third (and last) station of the first acquisition, so that the first acquisition consists of unenhanced images, and the arterial phase would occur at the beginning of the second acquisition. A total of seven acquisitions are iteratively performed, for a total acquisition time of around 5 min.

DCE MRI allows the extraction of time–signal intensity curves in both diffuse and focal marrow involvement areas, on a whole-body scale. Quantitative parameters of DCE MRI were found to correlate well with both microvessel density and serum markers of disease activity [34]. At diagnosis, increased contrast uptake was associated with a high infiltration degree [34,35]. Lin et al. showed that patients with at least 10% plasma cell infiltration on bone marrow aspiration had higher values of maximal percentage of bone marrow enhancement and steeper contrast agent wash-in slopes [22]. After treatment, disease progression or relapse might be easily detected in case of persistent abnormal maximal percentage of bone marrow enhancement and early enhancing focal lesions [6]. Moreover, relative signal enhancement is of prognostic significance for time to progression into symptomatic disease, in patients with smoldering MM [36], while increased bone marrow enhancement indicated shorter progression-free survival in patients with progressive MM [37].

Owing to the lack of standardization of image acquisition and analysis, DCE MRI is currently not recommended in a clinical routine. As stressed earlier regarding DW MRI [38], there is a need for recommendations on both data acquisition and analysis to enable DCE MRI to become robust, reproducible among centers, and ultimately be evaluated for potential integration into routine clinical practice.

## 3. ^18^F-FDG PET/MRI

**Figure 3:**
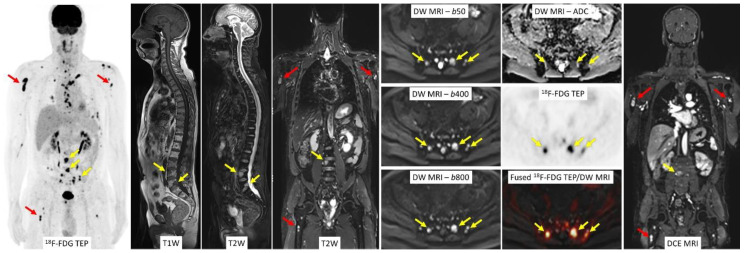
Hybrid PET/MRI provides simultaneous acquisition of morphological, functional, and metabolic information. Focal lesions localized in both the axial skeleton (**yellow** arrows) and the proximal appendicular skeleton (**red** arrows) are clearly depicted on the different images. ^18^F-FDG PET, DW MR, and DCE MR images provide combined information on the activity of the multifocal disease.

Hybrid PET/MRI provides simultaneous acquisition of morphological, functional, and metabolic information. PET/MRI allows the combination of the reference standard imaging modality for detecting bone marrow involvement (MRI), with the most recognized examination modality for prediction of both prognosis and treatment response (^18^F-FDG PET), and thus appears to be a promising imaging tool in the setting of MM (Figure 3).

**Figure 4:**
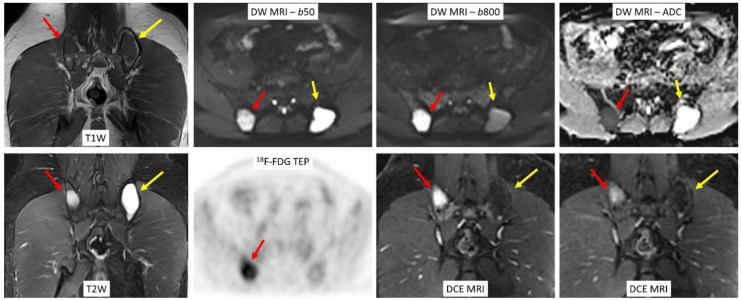
Coronal T1-weighted spin-echo and fat-suppressed T2-weighted images of the pelvis shows bilateral focal lesions in the right and left iliac bones, hypointense on T1- and hyperintense on T2-weighted images. Focal lesion on the right iliac bone (**red** arrows) appears to be metabolically active on transverse ^18^F-FDG PET image, has high signal intensity on both low and high *b* value DW images, higher ADC value than surrounding, and apparently normal bone marrow. Coronal DCE MR image shows an early and intense contrast-enhancement after contrast media administration, consistent with an active lesion. On the other hand, focal lesion on the left iliac bone (**yellow** arrows) appears to be metabolically inactive, and with no restriction of diffusion. Weak and thin linear and peripheral enhancement can be seen after contrast media administration, which is consistent with an inactive lesion.

With regards to attenuation correction and semi-quantification, Sachpekidis et al. found equivalent performance and high SUV_max_ correlation, using either PET/CT or PET/MRI in both MM lesions and non-tumoral bone marrow [39]. ^18^F-FDG PET/MRI might improve focal lesions detection and initial staging in patients with MM [40], and might also be able to diagnose and localize residual disease activity, and therefore, might help guide treatment in patients who achieved a complete remission (Figure 4) [3]. Nonetheless, further studies are needed to specify both the optimal imaging protocol in both staging and post-treatment evaluation, especially the place of functional MR imaging, and standard hybrid features interpretation and significance, especially in case of discordance between modalities [4].

## 4. MRI Indications in Multiple Myeloma

The International Myeloma Working Group recently provided consensus recommendations on imaging in monoclonal plasma cell disorders. Although the superiority in sensitivity of both ^18^F-FDG PET/CT and whole-body MRI over whole-body CT is acknowledged, whole-body CT is recommended as the primary imaging method in most indications, owing to the risk of false-positive findings on MR images and to the more limited availability of MRI and PET/CT worldwide [5]. Whole-body MRI is the first recommended imaging technique, solely in patients with solitary bone plasmocytoma. In patients with high-risk MGUS, whole-body CT is recommended to rule out MM, whole-body MRI is an alternative in case of CT non-availability or CT equivocal findings. In newly diagnosed smoldering MM and MM, whole-body CT is also the first imaging technique recommended. despite the recognized prognostic significance of both MRI and PET/CT findings. This is explained by the absence of impact on therapy of such findings, and by the outstanding importance and accurate assessment of the extent of bone destruction. Whole-body MRI should be performed in case of either negative or inconclusive whole-body CT. This is also the case at relapse.

## 5. Conclusions

The introduction of new treatment strategies owing to the approval of several molecules in the last decade, enabled a significant increase in survival in patients with MM. In this context, imaging has an increasingly important role in the care of patients with MM patients for diagnosis, follow-up, and evaluation of both treatment response and lesion complications. Whole-body MRI represents the most sensitive imaging technique for the detection of bone marrow involvement in MM. Functional MRI further improves MRI performances for both diagnosis and treatment response assessment, owing to additional information on lesion vascularity and cellularity allowing detection of active myeloma lesions. Diffusion-weighted imaging was found to have the highest sensitivity for bone marrow involvement, and its systematic use is now recommended in clinical practice [3,7]. The results of such sequences need further validation, especially in treatment-response assessment. The recent introduction of MY-RADS recommendations is also an important step toward a systematic use in clinical routine, by allowing standardization of both acquisition protocols and image interpretation. Finally, hybrid PET/MRI imaging might act as the optimal imaging modality in MM, owing to the association of the reference standard imaging modality for detecting bone marrow involvement (MRI) and the most recognized examination modality for prediction of both prognosis and treatment response (^18^F-FDG PET) in a unique examination, providing one-stop-shop imaging for staging in a whole-body scale. One of the main challenges remain in the analysis of the potential impact of both whole-body MRI and PET/CT findings, on the therapeutic management of patients with MM at both diagnosis and relapse.
